# Site-1 protease–mediated cholesterol metabolism is essential for lymphatic development in mice

**DOI:** 10.1172/jci.insight.188637

**Published:** 2025-10-22

**Authors:** Yuji Kondo, Yizhi Jiang, Xin Geng, Jianhua Song, Summer Simeroth, J. Michael McDaniel, Pengchun Yu, R. Sathish Srinivasan, Lijun Xia

**Affiliations:** 1 Cardiovascular Biology Research Program, Oklahoma Medical Research Foundation, Oklahoma City, Oklahoma, USA.; 2 Institute for Glyco-core Research (iGCORE), Nagoya University, Furo-cho, Chikusa-ku, Nagoya, Japan.; 3 Department of Cell Biology and; 4 Department of Biochemistry and Physiology, University of Oklahoma Health Sciences Center, Oklahoma City, Oklahoma, USA.

**Keywords:** Metabolism, Vascular biology, Endothelial cells

## Abstract

Recent evidence suggests that cellular metabolism, including glycolysis and fatty acid synthesis in lymphatic endothelial cells (LECs), plays essential roles in developing functional lymphatic systems. Site-1 protease (S1P) proteolytically activates membrane-bound latent transcription factor sterol regulatory element-binding proteins (SREBPs), which are required to induce lipid biosynthesis. In this study, we generated mice with pan-endothelial or LEC-specific deficiency of either S1P or SREBP2. Mouse embryos with pan-endothelial deletion of S1P showed defective lymphatic vessel migration in skin and lymphedema, while their blood vasculature formation was relatively normal. Mice lacking S1P in LECs or SREBP2 in LECs exhibited chylous ascites, reduced lipogenic gene expression, and reduced VEGFR3 expression and progressively developed wasting, resulting in postnatal death by approximately 8 weeks of age. Additionally, mice with SREBP2 deletion in LECs exhibited dilated lacteal and mesenteric lymphatics and accumulation of lipids in the lacteal before weaning age, indicating apparent lymphatic malfunctioning. These data indicate that S1P-SREBP2–mediated cholesterol biosynthesis is pivotal in lymphatic vascular development. We also found that treating human dermal LECs with VEGF-C induced proteolytic activation of SREBP2 with concomitant phosphorylation of Akt and the expression of genes involved in cholesterol biosynthesis. Those effects were canceled out by treating the cells with an S1P inhibitor or SREBP inhibitor. These data demonstrate that the S1P/SREBP2 axis is critical in VEGF-C/VEGFR3 mitogenic signaling in LECs.

## Abstract 2

Site-1 protease is critical for lymphatic vascular development

## Graphical abstract

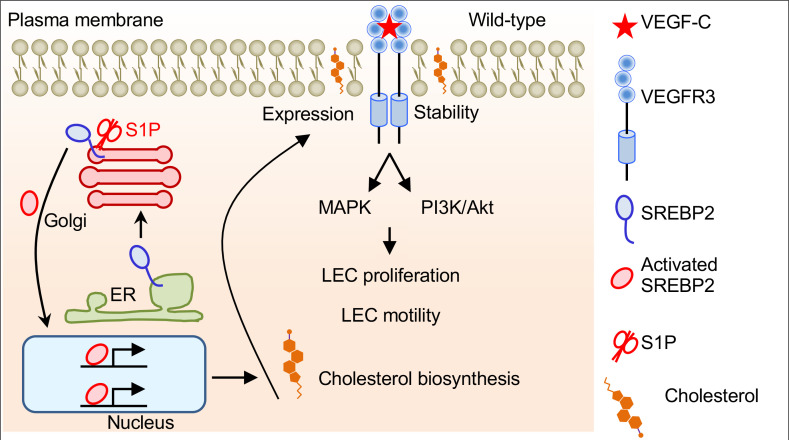

## Introduction

Cellular metabolism, converting nutrients such as glucose, fatty acids, or specific amino acids into biomass and energy, is essential for cell proliferation, structure maintenance, and function. New evidence has indicated that cellular metabolism is uniquely required for endothelial biology, including vascular sprouting, proliferation, and differentiation (1–11).

Unlike nutrients such as glucose and fatty acids, cholesterol is not used as a carbon source for energy production. Instead, cholesterol is essential for the biosynthesis of bile acid and specific vitamins and steroid hormones, as well as being a component of the plasma membrane of all cells (12–14). Cholesterol is absorbed from the small intestine through the Niemann-Pick C1-like 1 transporter, or de novo–biosynthesized in the liver, and excess blood cholesterol is excreted into the bile (15, 16). At the molecular level, cholesterol biosynthesis is elegantly regulated. Upon cholesterol deprivation, endoplasmic reticulum (ER) membrane-bound sterol regulatory element-binding protein 2 (SREBP2), encoded by sterol regulatory element-binding transcription factor 2 (*SREBF2*), translocates to Golgi membrane via a COP-II vesicle along with SCAP escorting protein and undergoes proteolysis by sequential actions of Golgi-localized site-1 protease (S1P) and site-2 protease (S2P) (17–20). The cleaved SREBP2 is liberated from the Golgi membrane and travels to the nucleus to induce gene expression in cholesterol biosynthesis (17, 21). Regarding lipogenesis, S1P also proteolytically activates SREBP1, which mainly induces genes required for triacylglycerol biosynthesis (21, 22). Until now, the role of S1P/SREBP2 axis–mediated cholesterol biosynthesis in the development of vasculature has remained elusive.

Here, we report that the mice lacking endothelial S1P, encoded by the gene named membrane-bound transcription factor peptidase, site 1 (*MBTPS1*), or SREBP2 exhibited defective lymphatic vascular development, demonstrating an essential role of the S1P/SREBP2 axis in vascular development. Our results reveal that S1P-SREBP2–mediated cholesterol biosynthesis regulates cholesterol content in the lipid raft of the plasma membrane of lymphatic endothelial cells (LECs) to control the VEGFR3 signaling during lymphangiogenesis.

## Results

### Mice lacking S1P in LECs show defective lymphatic vascular development.

Prior studies revealed the vital role of S1P in embryonic development by analyzing constitutive global S1P-deficient mice (23). To gain insights into the function of S1P in the vascular system, we generated mice with constitutive deletion of S1P in endothelium (*Mbtps1^fl/fl^ Tie2Cre*, EC *Mbtps1^–/–^*) using the well-established *Tie2Cre*-transgenic mice (24), which also mediates deletion in the hematopoietic lineage. We found that EC *Mbtps1^–/–^* mice showed embryonic lethality at embryonic day 9.5 (E9.5) (Supplemental Figure 1; supplemental material available online with this article; https://doi.org/10.1172/jci.insight.188637DS1). As EC *Mbtps1^–/–^* mice had S1P deficiency in both endothelial and hematopoietic cells, we generated mice with hematopoietic deletion of S1P (*Mbtps1^fl/fl^ VavCre*, HC *Mbtps1^–/–^*) by breeding *Mbtps1^fl/fl^* female mice with *Mbtps1^fl/wt^* *VavCre* male mice, which mediates deletion specifically in hematopoietic cells. HC *Mbtps1^–/–^* mice exhibited no substantial abnormal phenotypes, indicating that S1P has no critical role in the development and function of hematopoietic cells. This result also demonstrates the essential role of endothelial S1P in embryonic development.

To bypass the early embryonic lethality of the EC *Mbtps1^–/–^* mice, we generated mice with tamoxifen-inducible deletion of S1P (*Mbtps1^fl/fl^ Cdh5CreER^T2+^*, iEC *Mbtps1^–/–^*). *Cdh5CreER^T2^* is known to mediate the deletion of a floxed gene primarily in vascular endothelial cells. Blood vasculature formation during E10.5 to E12.5 in the hindbrain is a well-studied model for angiogenesis (25). We treated the *Mbtps1^fl/fl^ Cdh5CreER^T2^* pregnant dam with tamoxifen starting at E8.5, and the embryos were dissected at E12.5 (Supplemental Figure 2, A–C). We found relatively normal directional blood vasculature formation toward the subventricular zone (Supplemental Figure 2, D and E), indicating that S1P is not critical for angiogenesis in the hindbrain during this stage of development.

**Figure 1:**
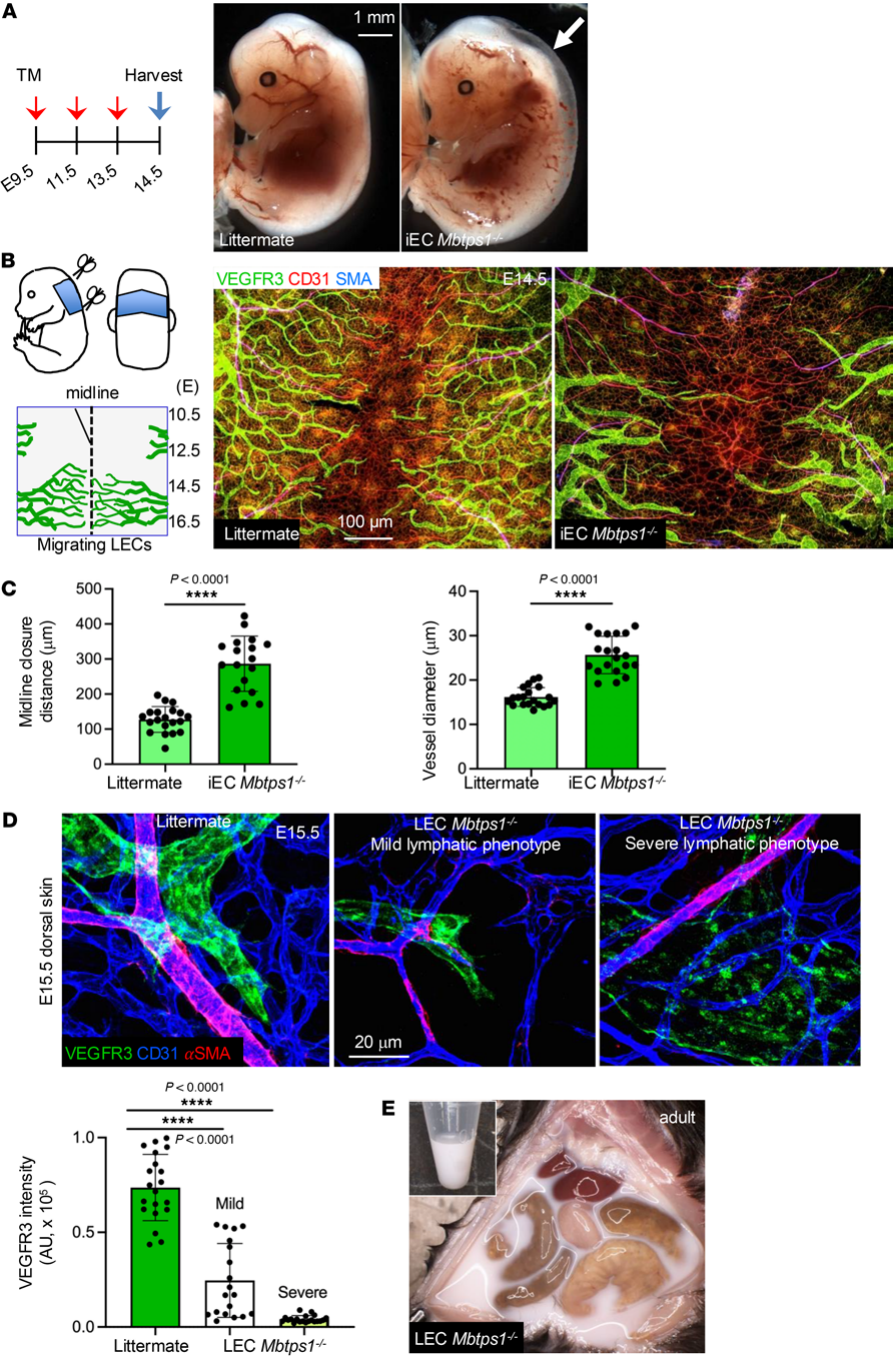
Mice lacking S1P in LECs show defective lymphatic phenotypes. (**A**) The diagram on the left depicts the tamoxifen-induced deletion of S1P to generate iEC *Mbtps1^–/–^* mice. Gross images on the right show embryos at E14.5 (*N* > 25 embryos in each genotype). Arrow shows lymphedema. (**B**) Left: Diagrams showing the collection of fetal anterior dorsal skin for whole-mount immunostaining and the migration of LECs to the midline of the dorsal skin at different developmental stages. Right: Images of immunostained anterior dorsal skin of WT and iEC *Mbtps1^–/–^* mice at E14.5. Data represent at least 3 experiments. SMA, smooth muscle actin. (**C**) Quantification of the distance to the midline covered by lymphatic vessels (left) and the vessels’ diameters (right). Based on the blood vascular patterning, comparable regions among samples (*n* > 5 images in each genotype) were selected as regions of interest for quantification. Midline closure and vessel diameter were measured using ImageJ (NIH). Littermates, WT. Data represent mean ± SD; unpaired *t* test with Welch’s correction was performed for the statistical analysis. ****P < 0.0001. (**D**) Immunostaining of dermal lymphatic vessels (green), arterioles (red), and blood capillaries (blue) in WT and LEC *Mbtps1^–/–^* mice at E15.5 (top). Quantification of VEGFR3 intensity normalized to the vessel area (bottom, *n* > 5 images in each genotype) using ImageJ. Data represent mean ± SD. One-way ANOVA was performed for the statistical analysis. ****P < 0.0001. (**E**) Chylous ascites in LEC *Mbtps1^–/–^* mice (*N* > 10 mice). Littermates, WT.

To further determine the vascular development of iEC *Mbtps1^–/–^* embryos, we induced the S1P deletion by tamoxifen injection into the pregnant dam at E9.5, E11.5, and E13.5 (Figure 1A). This time point allowed us to examine the development of both blood and lymphatic vascular systems in the dorsal skin of E14.5 embryos (Figure 1B). At E14.5, we noticed that approximately 30% of iEC *Mbtps1^–/–^* embryos had edema at the dorsal side (Figure 1A), a sign of defective lymphatic function, relative to wild-type littermate controls (mice with floxed alleles, hereafter, littermate or WT). Immunostaining of CD31 (pan-endothelial marker) and VEGFR3 (lymphatic endothelial marker) of the dorsal skin revealed that, compared with that of blood capillaries (defined as CD31^+^), migration of lymphatic capillaries (defined as CD31^+^VEGFR3^+^) to midline was severely impaired, supporting a primary role of S1P in lymphangiogenesis (Figure 1, B and C).

To study the intrinsic role of S1P in LECs, we generated mice with LEC-specific deletion of S1P (*Mbtps1^fl/fl^ Lyve1Cre^+^*, hereafter, LEC *Mbtps1^–/–^*). The mice showed defective lymphatic phenotypes characterized by a substantial reduction of VEGFR3-expressed LECs in the skin (Figure 1D) and chylous ascites (Figure 1E). Of note, most of the mice succumbed by 8 weeks of age. These data suggest that S1P is essential for the development of the lymphatic vascular system in mice and vital for their survival.

### LEC-specific SREBP2 deletion in mice causes postnatal lethality.

**Figure 2:**
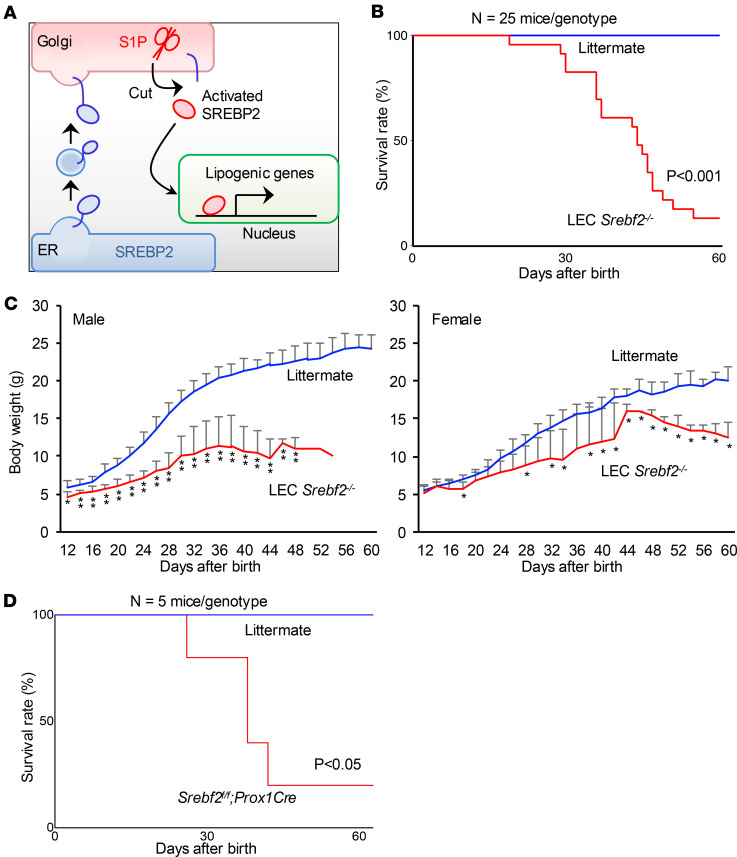
LEC-specific SREBP2 deletion in mice causes postnatal lethality. (**A**) Diagram depicting the role of S1P/SREBP2 axis in cholesterol biosynthesis. S1P proteolytically activates SREBP2, which in turn enters the nucleus to turn on the transcription of lipogenic genes. (**B**) Survival rate of LEC *Srebf2^–/–^* mice and littermate control (*N* = 25 mice in each group). (**C**) Changes in body weight of both male and female LEC *Srebf2^–/–^* mice (*N* > 10 mice in each group). (**D**) Survival rate of *Srebf2^fl/fl^ Prox1Cre* mice (*N* = 5 mice in each group). Kaplan-Meier curves were calculated for day-to-event endpoints, with differences tested by log-rank test. Body weight curves were created until day 60, with differences tested by unpaired *t* test on each time point. Data represent mean ± SD. *P < 0.05, **P < 0.01.

S1P proteolytically activates latent membrane-bound transcription factors at Golgi. Thus, lymphatic developmental defect in S1P-deficient mice is most likely caused by the defective cleavage of its substrates in LECs, such as SREBP2, the master transcription factor for cholesterol biosynthesis. Recent growing evidence regarding the importance of endothelial metabolism in lymphatic development and function motivated us to test if the cholesterol biogenesis pathway in LECs is involved in lymphatic development (Figure 2A). We generated mice with LEC-specific deletion of SREBP2 (*Srebf2^fl/fl^ Lyve1Cre^+^*, hereafter, LEC *Srebf2^–/–^*). LEC *Srebf2^–/–^* mice died postnatally by 8 weeks of age, resembling LEC *Mbtps1^–/–^* mice, indicating that the S1P/SREBP2 axis is essential for LECs and vital for survival (Figure 2B). Both male and female mutant mice showed growth retardation, characterized by smaller gain of body weight (Figure 2C). Because global deletion of SREBP2 in mice was reported to cause embryonic lethality (19), the postnatal lethality of LEC *Mbtps1^–/–^* mice and LEC *Srebf2^–/–^* mice is likely to be caused by loss of function of S1P or SREBP2 in LECs. To validate our observation, we used another LEC-specific Cre mouse, *Prox1Cre*. We found that *Srebf2^fl/fl^ Prox1Cre* mice phenocopied LEC *Srebf2^–/–^* mice by showing postnatal death (Figure 2D). These data demonstrate that the phenotype of LEC *Srebf2^–/–^* mice is caused by intrinsic loss of SREBP2 in LECs.

### Mice with LEC-specific SREBP2 deletion show dilated lacteals and low plasma triglyceride.

**Figure 3:**
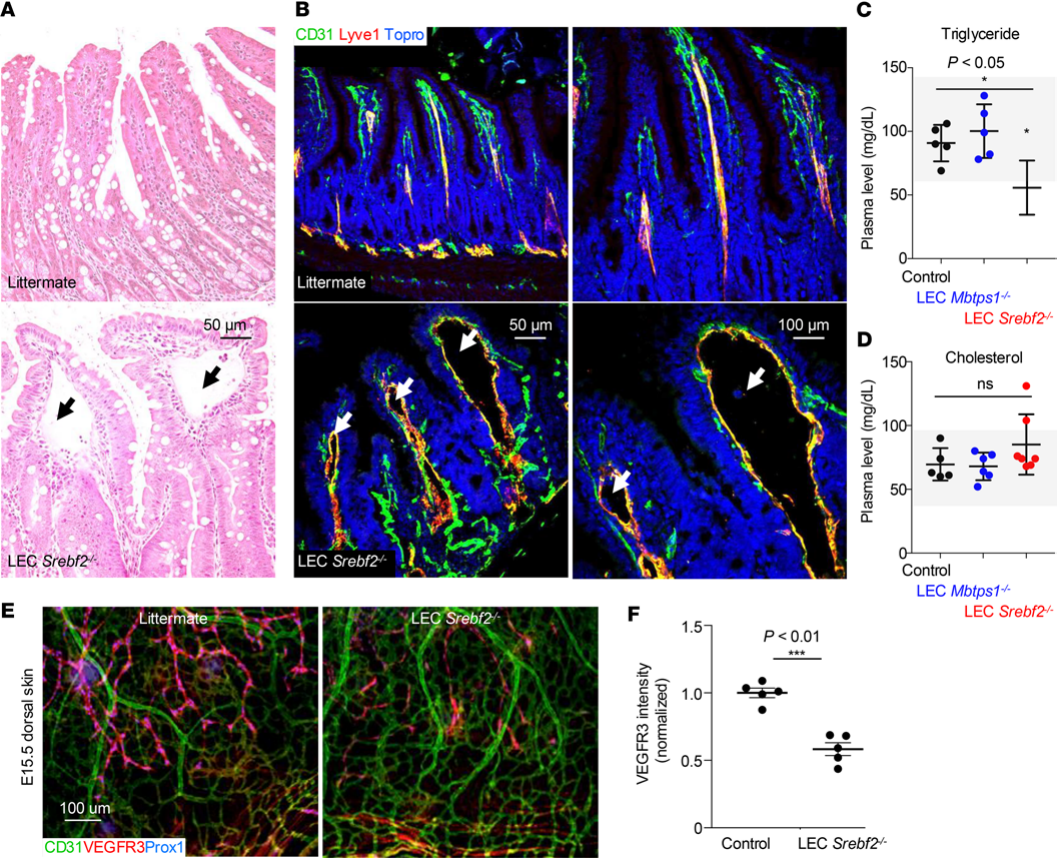
Mice with LEC-specific SREBP2 deletion show dilated lacteals. (**A**) Representative images of hematoxylin and eosin–stained sections of the ileum from littermate control and LEC *Srebf2^–/–^* mice (6 weeks of age). Arrows mark dilated lacteals. (**B**) Representative images of immunostaining of endothelial cells (green) and lymphatic vessels (red) in littermate control and LEC *Srebf2^–/–^* mice. Topro is a nucleus marker. Arrows indicate dilated lacteals. (**C** and **D**) Levels of plasma cholesterol and triglyceride. Each dot represents an individual mouse (*N* = 5–7). Gray areas mark the normal range. Data represent mean ± SD. One-way ANOVA was performed for the statistical analysis. *P < 0.05. (**E**) Whole-mount immunostaining of dermal lymphatics (blue), blood vessels (green), and VEGFR3 (red) in WT and LEC *Srebf2^–/–^* mice at E15.5. (**F**) Quantification of VEGFR3 intensity of **E** (*n* = 5 images/genotypes). Data represent mean ± SD. An unpaired *t* test was performed for the statistical analysis. ***P < 0.01.

To study the role of the S1P/SREBP2 axis in the postnatal lymphatic vascular system, we analyzed the structures of lymphatic capillaries in intestinal villi, called lacteals, in the jejunum and ileum of the surviving LEC *Srebf2^–/–^* mice by hematoxylin and eosin staining and found abnormally dilated lacteals in these mice (Figure 3A). This dilation of lacteals was also verified by immunostaining of CD31 (pan-endothelial marker) and Lyve1 (lymphatic endothelial marker) of the same tissues (Figure 3B). These data demonstrate that the S1P/SREBP2 axis is indispensable for normal lymphatic vascular development in embryonic and adult stages. We did not observe inflammatory cell infiltrates near the dilated lacteals in the small intestine (Figure 3A), suggesting LEC intrinsic defect caused the abnormal lacteal structure. Chylous ascites were a prominent feature of abnormal leakiness of lymphatic vasculature in the intestine in LEC *Srebf2^–/–^* mice. As expected, we observed lower plasma triglyceride levels in LEC *Srebf2^–/–^* mice than in littermate control mice (Figure 3C). Interestingly, although LEC *Mbtps1^–/–^* mice also exhibited chylous ascites (Figure 1E), they had normal plasma triglyceride levels. Plasma cholesterol levels in both mutant mice were comparable to control mice (Figure 3D). Like LEC *Mbtps1^–/–^* mice (Figure 1D), we observed a considerable reduction of VEGFR3 expression in Prox1^+^ lymphatic vasculature in the embryonic dorsal skin of LEC *Srebf2^–/–^* mice at E15.5 (Figure 3, E and F).

### Mice with LEC-specific S1P or SREBP2 deletion show dilated lacteals before weaning.

**Figure 4:**
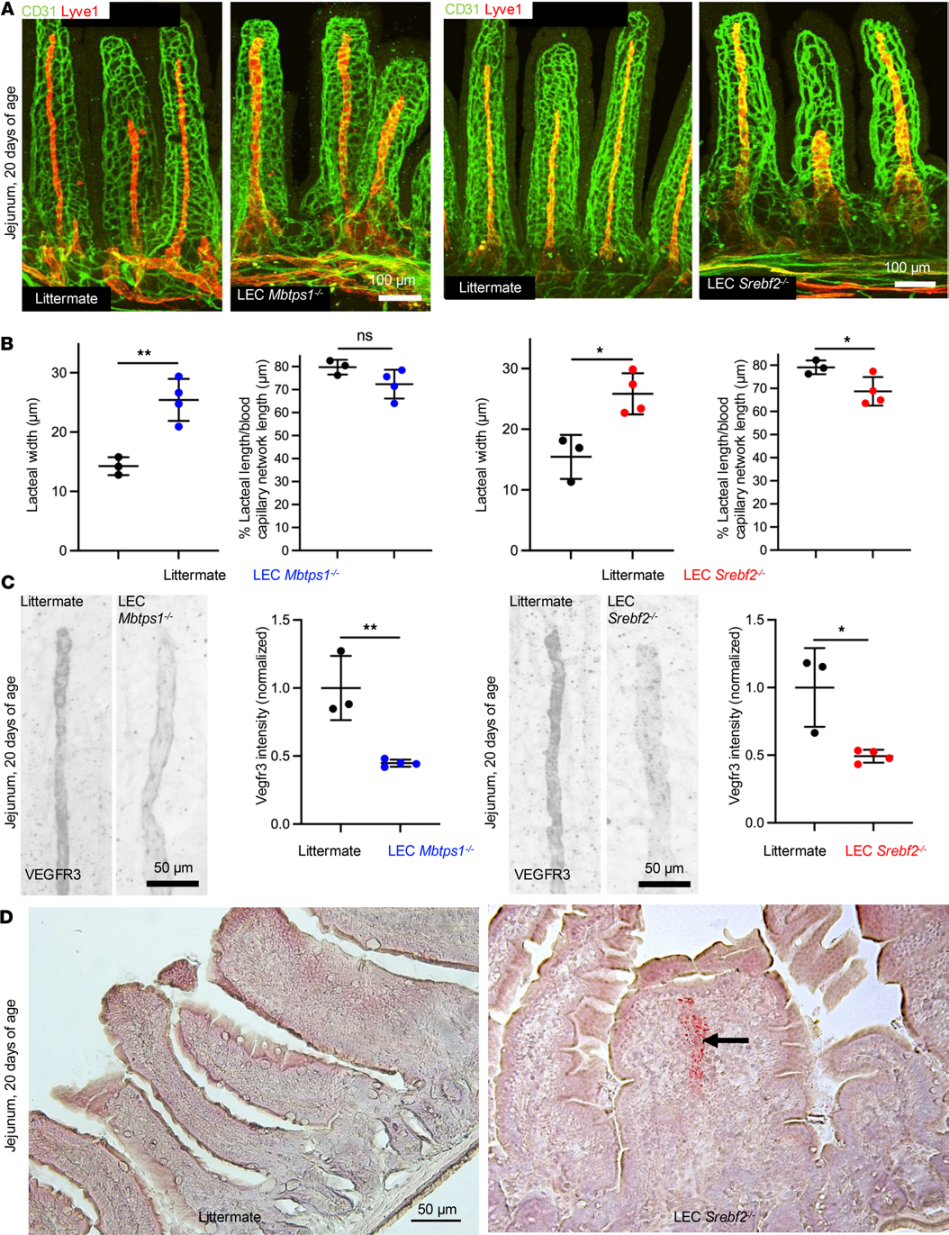
Mice with LEC-specific deletion of MBTPS1 or SREBP2 show dilated lacteals. (**A**) Representative whole-mount IF images of endothelial cells (green) and lymphatic vessels (red) in sections of jejunum from littermate control and LEC *Mbtps1^–/–^* mice (20 days of age). (**B**) Quantification of the length and width of the lacteal of littermate controls and LEC *Mbtps1^–/–^* mice. (**C**) Whole-mount IF images of VEGFR3 of lacteals of jejunum from WT littermates and LEC *Mbtps1^–/–^* or LEC *Srebf2^–/–^* mice (20 days of age). Fluorescence intensity of VEGFR3 was quantified using ImageJ and normalized to the average intensity of control lacteals. (**D**) Representative images of Oil Red O staining of jejunum section of LEC *Srebf2^–/–^* mice and littermates (20 days of age). Arrow marks Oil Red O–positive lacteals. *N* = 3 for control littermates; *N* = 4 for each mutant genotype respectively. A total of 12 lacteals from each animal were analyzed. Each dot represents 1 animal on the graph. The graphs were plotted as mean ± SD. An unpaired *t* test was performed for the statistical analysis. *P < 0.05; **P < 0.01.

To address whether the intestinal lymphatic defects occurred when mice were fed on milk, we examined both LEC *Mbtps1^–/–^* mice and LEC *Srebf2^–/–^* mice at postnatal day 20, right before weaning. Representative whole-mount immunofluorescence confocal images of jejunum samples showed substantially dilated lacteals of both LEC *Mbtps1^–/–^* and LEC *Srebf2^–/–^* mice relative to littermate controls (floxed alleles, Cre negative) (Figure 4, A and B, and Supplemental Videos 1 and 2). Immunostaining of jejunum lacteals with anti-VEGFR3 revealed substantially reduced fluorescence intensity of VEGFR3 in mutant lacteals after normalization to the average intensity of littermate control lacteals (Figure 4C). In addition, LEC *Mbtps1^–/–^* and LEC *Srebf2^–/–^* mice exhibited enlarged mesenteric collecting lymphatic vessels, as demonstrated by representative whole-mount images of mesenteric lymphatic vessels, in comparison with littermates (Supplemental Figure 3). Furthermore, we detected increased Oil Red O staining in the lacteals of LEC *Srebf2^–/–^* mice compared with littermates, suggesting abnormal accumulation of lipids in the mutant intestinal lymphatic system (Figure 4D).

To check if lymphatic vessels in other organs were affected at this age, we examined the dorsal ear skin dissected from both LEC *Mbtps1^–/–^* and LEC *Srebf2^–/–^* mice and wild-type littermates. Our analyses revealed only a modest reduction of lymphatic microvascular density in the LEC *Mbtps1^–/–^* dorsal ear relative to the littermates (Supplemental Figure 4A). However, collective lymphatic vessels in both LEC *Mbtps1^–/–^* and LEC *Srebf2^–/–^* dorsal ear had no substantial abnormalities (Supplemental Figure 4B). These data indicate that the lymphatic defects are mainly present in the small intestine of LEC *Mbtps1^–/–^* and LEC *Srebf2^–/–^* mice.

### Mice with deletion of SREBP2 in LECs show hypotriglyceridemic and wasting syndrome.

**Figure 5:**
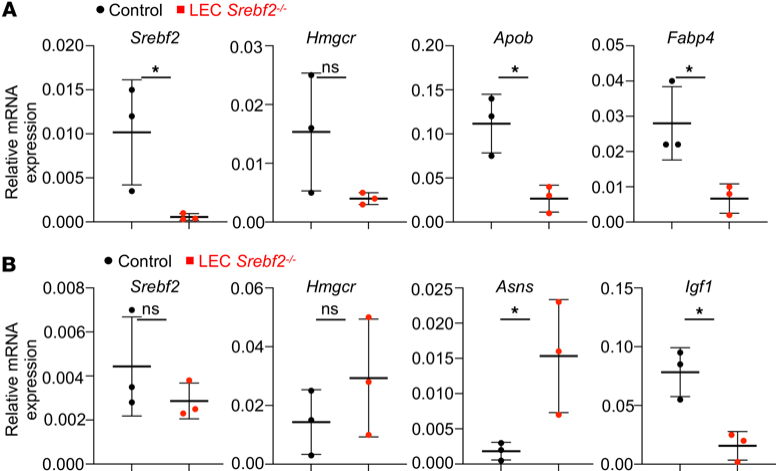
Mice with deletion of SREBP2 in LECs show hypotriglyceridemic and wasting syndrome. (**A**) qPCR results of *Srebf2*, *Hmgcr*, *Apob*, and *Fabp4* in the small intestine from control and LEC *Srebf2^–/–^* mice (4–6 weeks of age, *N* = 3 mice/genotype). (**B**) qPCR of *Srebf2*, *Hmgcr*, *Asns*, and *Igf1* in the livers from control and LEC *Srebf2^–/–^* mice (4–6 weeks of age, *N* = 3 mice/genotype). The graphs were plotted as mean ± SD. An unpaired *t* test was performed for the statistical analysis. *P < 0.05.

Poor absorption of dietary lipids via lacteal into blood circulation causes growth retardation and malnutrition (26). As expected, quantitative reverse transcription PCR (qPCR) results showed reduced gene expression of lipid carrier proteins (*Apob* and *Fabp4*) in the small intestine from LEC *Srebf2^–/–^* mice (Figure 5A). As an indication of malnutrition and resultant wasting syndrome, elevated asparagine synthetase (*Asns*) and reduced insulin growth factor 1 (*Igf1*) expression in livers from LEC *Srebf2^–/–^* mice were found (Figure 5B), which resemble *Plagl2*-knockout mice in which lacteals in jejunum are also dilated (26). These data suggest that lack of SREBP2 in LECs causes defective lymphatics, poor lipid bioavailability, and lethal malnutrition. Both the LEC *Mbtps1^–/–^* and the LEC *Srebf2^–/–^* mice died postnatally around 8 weeks of age. This postnatal lethality is presumably caused by poor absorption of triglyceride from the intestine and resultant wasting syndrome. The reason plasma triglyceride in LEC *Mbtps1^–/–^* mice was comparable to that of control mice is not apparent (Figure 3C). qRT-PCR with RNAs isolated from sorted LECs from the small intestine using flow cytometry verified a global reduction of lipogenic gene expression in LEC *Mbtps1^–/–^* mice (Supplemental Figure 5).

### VEGF-C/VEGFR3 signaling is S1P dependent.

**Figure 6:**
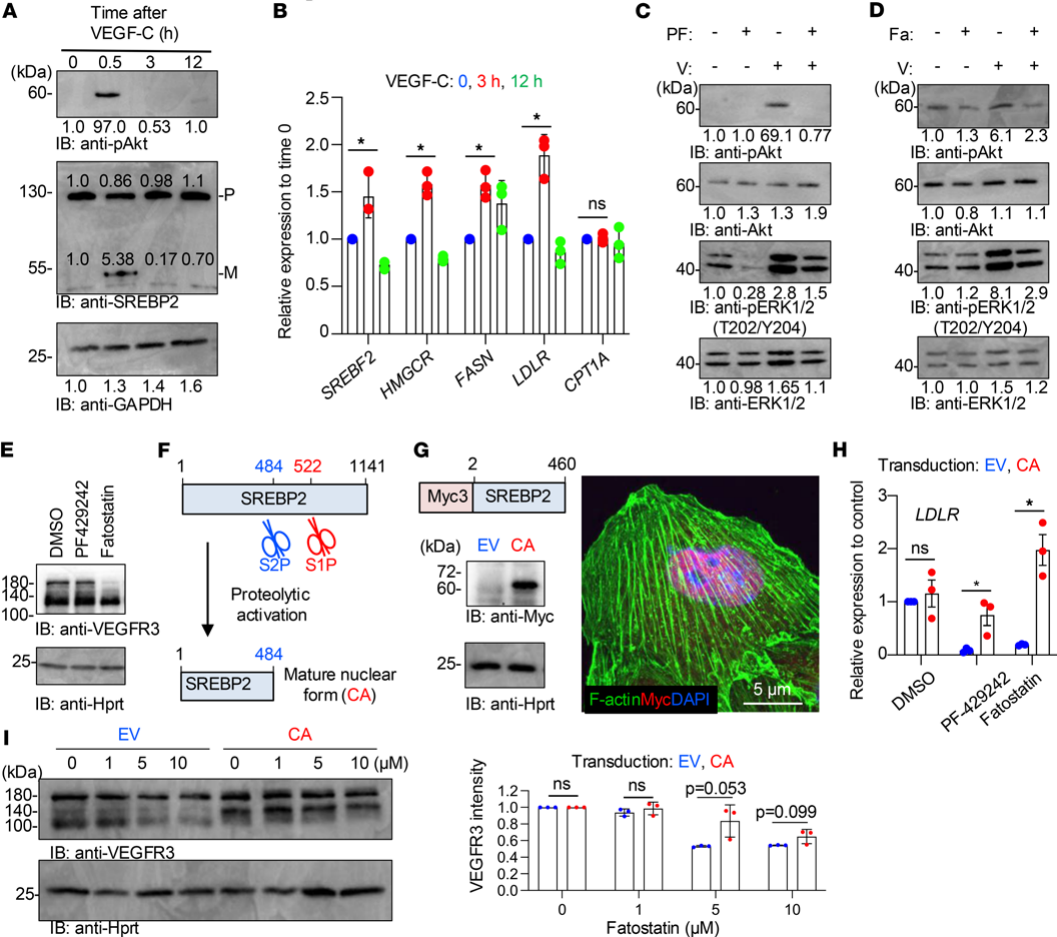
VEGF-C/VEGFR3 signaling is S1P dependent. (**A**) Western blotting of phosphorylated Akt and SREBP2 in cultured human dermal LECs stimulated with 10 ng/mL VEGF-C. P, precursor; M, matured (i.e., cleaved). (**B**) Quantitative reverse transcription PCR (qRT-PCR) of lipogenic genes in the cultured cells treated with VEGF-C. The RNAs were isolated at 0, 3 and 12 hours after VEGF-C stimulation. Data represent mean ± SD. One-way ANOVA was performed for the statistical analysis. *P < 0.05. *N* = 3. (**C**) Western blotting of Akt and ERK1/2 in the cultured cells stimulated with VEGF-C (V) with the pretreatment of S1P inhibitor (PF; 5 μM PF-429242, 30 minutes) in **C** or SREBP inhibitor (Fa; 5 μM fatostatin, overnight) in **D**. (**E**) Western blotting of VEGFR3 when treated with S1P inhibitor or SREBP inhibitor (5 μM, overnight). Hprt is a loading control. (**F**) Schematic illustration of proteolytic activation of SREBP2 by sequential actions of S1P and S2P. Numbers denote amino acids. (**G**) Protein structure of constitutive active SREBP2 (CA) (top). CA is detected by Western blotting (bottom, left) and immunofluorescence using anti-Myc antibody (bottom, right). (**H**) *LDLR* gene expression was monitored by qRT-PCR as an indicator of SREBP2 activity. The graph was plotted as mean ± SD. Two-way ANOVA was performed for the statistical analysis. *P < 0.05. *N* = 3. (**I**) Western blotting of VEGFR3 expression when treated with S1P inhibitor or SREBP inhibitor (1, 5, 10 μM, overnight) with concomitant expression of constitutive active SREBP2 (CA) (left). Empty vector (EV) was used as a control. Quantification of VEGFR3 intensity of blots (right). The graph was plotted as mean ± SD. Two-way ANOVA was performed for the statistical analysis. *N* = 3. Data represent at least 3 experiments. Numbers underneath each blot indicate quantification of band intensity.

VEGFR3 and its cognate ligand VEGF-C transduce mitogenic signaling in LECs (27–29). Of note, LEC migration and proliferation were severely affected, and expression of VEGFR3 was downregulated in S1P-deficient LECs (Figure 1, B–E, and Figure 4C). Thus, we hypothesized that the S1P/SREBP2 axis was required for mitogenic VEGFR3 signaling. To test this, we stimulated primary cultured human dermal LECs (hLECs) with VEGF-C. We found that VEGF-C stimulation induced cleavage of SREBP2 within 30 minutes with a concomitant increase in phosphorylated Akt in hLECs (Figure 6A). As expected, the expression of genes for cholesterol synthesis, such as *HMGCR*, *FASN*, and *LDLR*, was upregulated upon VEGF-C stimulation, suggesting that VEGF-C/VEGFR3 signaling induced S1P-SREBP2–dependent cholesterol biogenesis in LECs (Figure 6B). Importantly, VEGF-C–mediated phosphorylation of Akt and ERK1/2 was impaired in the presence of PF-429242, an S1P inhibitor, and fatostatin, an SREBP inhibitor, respectively (Figure 6, C and D). We also observed that SREBP inhibition greatly reduced VEGFR3 expression in hLECs, albeit with little effect by S1P inhibition (Figure 6E). Next, to test the reliability of the SREBP inhibitor, we tested if forced expression of the N-terminal myc3-tagged mature form of SREBP2, a constitutively active (hereafter, CA) nuclear form, normalized SREBP inhibition–mediated VEGFR3 reduction (Figure 6, F and G). Normalization of altered LDLR expression mediated by either S1P or SREBP inhibition was verified by forced expression of CA form compared with empty vector (Figure 6H). As expected, forced expression of CA form maintained VEGFR3 levels even in the presence of SREBP inhibitor in a dose-dependent manner (Figure 6I). Together, these data indicate that the S1P/SREBP2 axis plays a critical role in LEC mitogenic signaling, which could explain the observed lymphatic malformation in the S1P-SREBP2–defective mice. We found reduced intensity of VEGFR3 immunostaining in hLECs treated with methyl-β-cyclodextrin. This compound limits the availability of cellular cholesterol (Supplemental Figure 6).

## Discussion

The function of S1P has been intensively studied for many decades, with focus on its roles in unfolded protein response and lipid biosynthesis (30). S1P carries out these functions by proteolytic activating of membrane-bound latent transcription factors, such as SREBPs and ATF6 (31, 32). GlcNAc-1-phosphotransferase is an exceptional S1P substrate as a non–transcription factor and is required for lysosome biogenesis via mannose-6-phosphate modification of lysosomal hydrolases (33). Recently, we reported a pediatric patient with an amorphic and a hypomorphic mutation in *MBTPS1* (30). The unique combination of these mutations results in reduced activity of S1P, which is associated with several developmental defects. Among these defects, we found that the patient exhibited abnormal plasma lipid levels, indicating, for the first time, the requirement of S1P for lipid metabolism in humans (30). The S1P patient we discovered has sufficient residual function of S1P to bypass the developmental lethality and exhibit abnormalities in selected tissues, suggesting a differential requirement of S1P function in different cell types. Since the complete loss of function of S1P is most likely lethal in humans, mice with cell-specific or inducible deletion of S1P are needed to reveal the unique function of S1P in specific cell types or organs.

In this study, we discovered a role of endothelial S1P on lymphatic development and function by analyzing cell type–specific S1P-deficient mice. Inducible deletion of S1P in endothelial cells during midgestation demonstrated that lymphatic vasculature was profoundly affected when compared with blood vasculature, suggesting a prominent role of S1P in lymphatic development (34). This result was validated by the observation that LEC-specific S1P-deficient mice (LEC *Mbtps1^–/–^*) had reduced VEGFR3 expression, dilated lacteals, and chylous ascites formation (35), demonstrating an essential function for S1P in lymphatic vascular development. The primary sequence of S1P is highly conserved among species ranging from fish to humans, suggesting its essentiality in living animals (30). DEPMAP database also indicates that deletion of S1P in many cancer cell lines causes pause in cell growth or death (36). Global deletion of S1P in mice leads to embryonic lethality at midgestation (23). In this study, we found that LEC-specific deletion of S1P or SREBP2 causes postnatal lethality. These data demonstrate that the S1P/SREBP2 axis has an indispensable role in vivo (23, 30).

S1P deficiency most likely causes global alteration of the transcriptome in LECs because of defective cleavage of membrane-bound transcription factors. VEGFR3 is a tyrosine kinase receptor expressed on LECs. It transduces Ras-Raf-MEK-ERK mitogenic signaling on the plasma membrane upon binding a cognate ligand, VEGF-C, during development and postnatally. Given that VEGF-C treatment of hLECs caused S1P-dependent phosphorylation of mitogen-activated protein kinase (MAPK) cascade, proteolysis of SREBP2, and induction of gene expression that is required for cholesterol biosynthesis, VEGFR3 is one of the most likely affected molecules upon loss of the S1P/SREBP2 axis. A possible mechanism is that Ras needs to be anchored underneath the plasma membrane via lipidation (37). Its lipidation is affected upon loss of S1P, leading to defective VEGFR3/MAPK signaling. VEGFR3 has been reported to exist in 3 molecular forms (38), and we observed corresponding bands in our blots. The 190 and 125 kDa forms localize to the cell surface (39), and their levels were reduced by SREBP inhibition in a dose-dependent manner (Figure 6I). In contrast, CA SREBP2 increased the 170 kDa form. These findings suggest that S1P/SREBP2 signaling supports VEGFR3 translation and cell surface expression. S1P also regulates collagen secretion via ER stress transducers like OASIS and BBF2H7, critical for lymphatic development in zebrafish (40). Our results indicate that SREBP2-driven cholesterol biosynthesis is likewise essential for lymphatic development. Since S1P deficiency affects multiple pathways, including SREBPs and ER stress responses, this likely explains the phenotypic differences between S1P and SREBP2 inhibition. Consistently, SREBP inhibition by fatostatin impairs lymphangiogenesis in cancer models (41), supporting our findings.

Interestingly, the development of blood vasculature is less affected than lymphatic vasculature in iEC *Mbtps1^–/–^* mice, which may be attributed to less importance or lower expression of S1P in blood endothelial cells compared with LECs. The level of endogenous S1P protein is known to be below the detection limit in imaging and Western blotting. Thus, we cannot directly address the aforementioned hypothesis. Nonetheless, the data presented here indicate a pivotal role of S1P in lymphatic development. Further studies are needed to elucidate the mechanism explaining how the S1P/SREBP2 axis contributes to VEGFR3 signaling.

## Methods

### Sex as a biological variable.

Sex as a biological variable was considered. The experiments used both sexes. Findings are expected to apply to both sexes.

### Mice.

All mice were maintained on a C57BL/6J congenic background. They were weaned at 21 days of age and fed a regular chow diet after weaning. Mice were genotyped routinely by PCR assay on genomic DNA isolated from tail clips. They were housed and maintained in a specific pathogen–free facility at the Oklahoma Medical Research Foundation.

Timed-mated females were obtained from natural matings by crossing males with females of breeding age. The presence of a copulatory plug was denoted as E0.5. For generation of mice with constitutive and inducible deletion of S1P in endothelial cells (*Mbtps1^fl/fl^ Tie2Cre^+^* and *Mbtps1^fl/fl^ Cdh5CreER^T2+^*), female *Mbtps1^fl/fl^* mice (provided by Jay Horton, University of Texas Southwestern Medical Center, Dallas, Texas, USA) were crossed with the male *Mbtps1^fl/wt^ Tie2Cre* transgenic mice (008863, Jackson Laboratory) or male *Mbtps1^fl/wt^ Cdh5CreER^T2^* transgenic mice. The *Cdh5CreER^T2^* transgenic mice were from the laboratory of Ralf Adams, currently also available through Taconic (23, 34, 42). Mice lacking S1P in LECs (*Mbtps1^fl/fl^ Lyve1Cre^+^* or *Mbtps1^fl/fl^ Prox1Cre^+^*) were generated by crossing female *Mbtps1^fl/fl^* mice with male *Mbtps1^fl/wt^ Lyve1Cre* or *Mbtps1^fl/wt^ Prox1Cre*-transgenic mice (43). For generation of mice with inducible deletion of SREBP2 in all endothelial cells (*Srebf2^fl/fl^ Cdh5CreER^T2+^*) or constitutive deletion in LECs (*Srebf2^fl/fl^ Lyve1Cre*^+^), female *Srebf2^fl/fl^* (provided by Jay Horton, University of Texas Southwestern Medical Center, Dallas, Texas, USA) (44) were bred with either male *Srebf2^fl/wt^ Cdh5CreER^T2^* or *Srebf2^fl/wt^ Lyve1Cre^+^* mice. The *Lyve1Cre* knockin was from the Jackson Laboratory (012601). To induce embryonic deletion of S1P, tamoxifen (MP Biomedicals) was dissolved in ethanol/sunflower oil (1:9) and administered intraperitoneally (500 μg per day) to the pregnant dam from E9.5 to E13.5 every other day (30). Littermates (*Mbtps1^fl/wt^* or *Mbtps1^fl/fl^ Srebf2^fl/wt^* or *Srebf2^fl/fl^*) treated with the same regimen were used as controls.

### Histology, immunostaining, and image acquisition.

Mouse embryos were collected and photographed at dissection. For histology, embryos were fixed in 10% formalin, and 5 μm paraffin-embedded sections were cut and stained with hematoxylin and eosin. For confocal microscopy of cryosections, mouse tissues were fixed in 4% paraformaldehyde (PFA) overnight at 4°C, washed in PBS, cryoprotected in 20% sucrose in PBS at 4°C overnight, embedded in 50% tissue freezing medium/50% OCT, cryosectioned (20–30 μm in thickness), and mounted for imaging with a ZEISS LSM710 microscopy system or a C2 Nikon confocal system.

Images were quantified blindly using ImageJ (NIH) by a lab data analyst. For vessel midline closure and vessel diameter, 20 readings were collected per genotype. These data were imported into GraphPad Prism, and a 2-tailed *t* test was performed to assess the significance between the samples. For the VEGFR3 staining intensity, the VEGFR3 channel was selected, and the threshold was adjusted to measure intensity across all 3 groups. The intensity data were imported into Prism, where a 1-way ANOVA was conducted to evaluate significance among the groups.

For whole-mount immunofluorescence (IF) of the intestine, mice were euthanized by asphyxiation followed by perfusion with PBS and 4% PFA. Subsequently, the gastrointestinal tract (from the stomach to the rectum) was dissected out, and the mesentery was removed from the gut. The jejunum and/or ileum were isolated, cleaned, opened, and pinned to the sylgard plate with villi facing up (45). The samples were subsequently fixed in 2% PFA overnight in the cold room and used for whole-mount IF using the modified iDISCO protocol previously described (46). After IF staining, the samples were postfixed in 4% PFA overnight in the cold room and washed with PBS. The intestinal villi were isolated as strips and mounted for imaging (45). The remaining small intestine and the mesentery were cleared for 2 days with Ce3D clearing solution (47). Cleared tissues were mounted on slides with spacers in Ce3D clearing solution and imaged with AX R confocal microscope (Nikon). 3D images were analyzed using Imaris software to generate volume and surface views.

Stitched images of the dorsal ear skin were acquired using a Nikon C2 confocal microscope. Comparable regions were chosen from the ears using Fiji ImageJ. To quantify the lymphatic vessel area, Lyve1-positive vessel area was measured utilizing the threshold function and then standardized to control. To quantify collecting lymphatic vessel length, SMA^+^ lymphatic vessels were traced by hand and measured for length before standardizing to control.

### Cell culture.

hLECs were purchased from PromoCell. hLECs were grown on tissue culture dishes precoated with 0.2% gelatin and maintained in EGM-2 EC Growth Medium-2 BulletKit (complete media, Lonza). All experiments were conducted using cells until passage 8. For the VEGF-C stimulation experiment, hLECs were precultured in 1:20 diluted EGM-2 EC Growth Medium-2 overnight and stimulated with 10 ng/mL of VEGF-C (PeproTech). Some hLECs were also pretreated with S1P inhibitor PF-429242 (5 μM, Cayman Chemicals) for 30 minutes. To limit the bioavailability of cellular cholesterol, hLECs were treated with 10 mM methyl-β-cyclodextrin for 0.5 hour before immunostaining of VEGFR3. In an overexpression study, HEK293T cells (CRL-3216, ATCC) (1 × 10^6^) cultured in 5% FBS in DMEM were plated in a 6-well plate. The next day, plasmids encoding VEGFR3 (1.5 μg) with either empty vector (pLVX-M-Puro, 1.5 μg) or constitutive active SREBP2 (1.5 μg), together with PEImax transfection reagent, were transfected. After 16 hours, cells were detached with trypsin-EDTA and replated in 4 wells of a 24-well plate. The next day, cells were treated with different doses of fatostatin in 0.5% FBS in DMEM for 24 hours. DMSO was used as a control.

### Antibodies and chemicals.

Antibodies against VEGFR3 (13-5988-82, eBioscience, or AF743, R&D Systems), CD31 (2H8, in-house), Alexa Fluor 647–conjugated CD31 (102515, BioLegend), Alexa Fluor 488–conjugated podoplanin (127405, BioLegend), Cy3-conjugated α-SMA (C6198, Sigma-Aldrich), Lyve1 (AF2125, R&D Systems; 11-034, AngioBio), GAPDH (sc-25778, Santa Cruz Biotechnology), phospho-Akt (Ser473, 9916, Cell Signaling Technology [CST]), total Akt (9916, CST), phospho-ERK1/2 (9911, CST), ERK1/2 (9911, CST), SREBP2 (MABS1988, MilliporeSigma), HPRT (ab109021, Abcam), FITC-conjugated CD11b (101205, BioLegend), Prox1 (11-002, AngioBio), Myc (sc-40, Santa Cruz Biotechnology), Ki67 (RM-9106-S0, Thermo Fisher Scientific), and PE-conjugated Ly6G (127607, BioLegend) were purchased. Tamoxifen was purchased from MP Biomedicals. Topro3 was purchased from Thermo Fisher Scientific. Phalloidin–Alexa Fluor 488 Conjugate (PA-3010) was purchased from Lonza.

### qRT-PCR.

Gene expression levels of total RNA extracted from tissues or cultured cells using TRIzol (Invitrogen) were analyzed by qPCR. RNA was converted to cDNA using the M-MLV Reverse Transcriptase system (Thermo Fisher Scientific). PCR analysis of cDNA was performed with the SYBR Green qPCR system using a CFX96 real-time system instrument (Bio-Rad) using primers listed in Supplemental Table 1.

### Flow cytometry.

Bone marrow from the tibia and femur was treated with ACK buffer to lyse red blood cells and stained with FITC–anti-CD11b and PE–anti-Ly6G antibodies on ice for 30 minutes. After washing with PBS once, cell fluorescence was analyzed by FACSCelesta flow cytometry.

### Blood analysis.

Peripheral blood from retro-orbital sinus was collected into EDTA-coated tubes. Plasma cholesterol and triglyceride were analyzed with IDEXX (IDEXX Laboratories) according to the manufacturer’s instructions.

### Western blotting.

Protein extracts from cultured LECs were prepared using a 1% Triton X-100–based cell lysis buffer. Lysates were applied to a 12% SDS-PAGE gel. Separated proteins were transferred onto an Immobilon-P membrane (MilliporeSigma). After blots were blocked, the membranes were incubated with antibodies and developed with ECL systems. Exposed x-ray films were scanned and analyzed using ImageJ software.

### Statistics.

Statistical analyses were performed using GraphPad Prism 9. Unless specified explicitly in the figure legends, an unpaired 2-tailed *t* test with Welch’s correction or a 1-way ANOVA with Dunnett’s post hoc test was used to determine the significance between experimental groups and the control groups. Significance of the Kaplan-Meier survival curves was determined using log-rank (Mantel-Cox) and Gehan-Breslow-Wilcoxon tests.

### Study approval.

Animal studies were conducted with protocols approved by the Institutional Animal Care and Use Committee of the Oklahoma Medical Research Foundation.

### Data availability.

Values for all data points in graphs are reported in the Supporting Data Values file.

## Author contributions

YK and LX designed the study and interpreted the data. YK, YJ, XG, JS, SS, JMM, and PY performed the experiments and analyzed the data. RSS contributed to data interpretation. YK, YJ, XG, and LX organized the data and wrote the manuscript. YK primarily contributed to the original submission and so is listed first. Both YJ and XG contributed substantially to the revisions.

## Supplementary Material

Supplemental data

Unedited blot and gel images

Supplemental video 1

Supplemental video 2

Supporting data values

## Version 1 10/22/2025

Electronic publication
