# Supplementary material for: Site-1 protease–mediated cholesterol metabolism is essential for lymphatic development in mice
Source: JCI Insight. 2025 Oct 22;10(20):e188637. doi: 10.1172/jci.insight.188637 (PMC12581667; doi:10.1172/jci.insight.188637)
Supplement: Supplemental data [file jciinsight-10-188637-s125.pdf]

Table 1: Primers for qRT-PCR

mouse	Forward (5' > 3')	Reverse (5' > 3')
Srebf2	GGGCTTCTTGGCTAGCTACT	CAAGGACTCCACCGCTCTTT
Hmgcr	TTGGCCTCCATTGAGATCCG	CTGCTCAGCACGTCCTCTTC
Apob	TTCCAGATTGCTAGGCTCCC	CTGGTAGGTATCACGGGCT
Fabp4	TCACCATCCGGTCAGAGAGT	TTCATCGAATTCCACGCCCA
Asns	CCTTTTATCAGGGGGCCTGG	CAGATGCCCGAACTGTCGTA
Igf1	AATCAGCAGCCTTCCAACTCA	GAGCTGGTGAAGGTGAGCAA

human	Forward (5' > 3')	Reverse (5' > 3')
SREBF2	ATGGGCAGCAGAGTTCCTTC	CGACAGTAGCAGGTCACAGG
HMGCR	CGATGCATAGCCATCCTGTA	GTGCTTGCTCTGGAAAGGTC
FASN	GGTCTTGAGAGATGGCTTGC	AATTGGCAAAGCCGTAGTTG
LDLR	TTCACTCCATCTCAAGCATCG	ACTGAAAATGGCTTCGTTGATG
CPT1A	GCAGCGTTCTTTGTGACGTT	AGGAGTGTTCAGCGTTGAGG

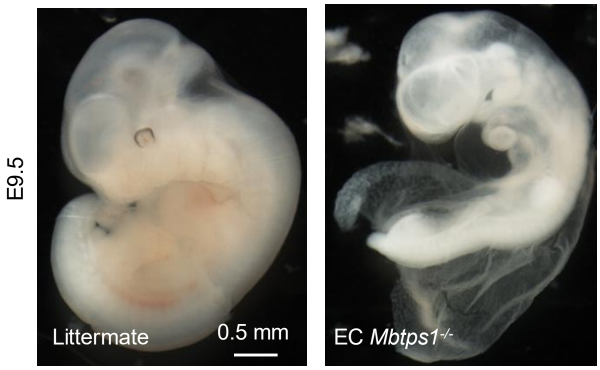

Supplemental Figure 1. Embryonic lethality of mice lacking endothelial- and hematopoietic-S1P (EC *Mbtps1*$^{-/-}$, n > 15 embryos in each genotype). All littermate controls are alive while all the EC *Mbtps1*$^{-/-}$ embryos are dead based on heart beating.

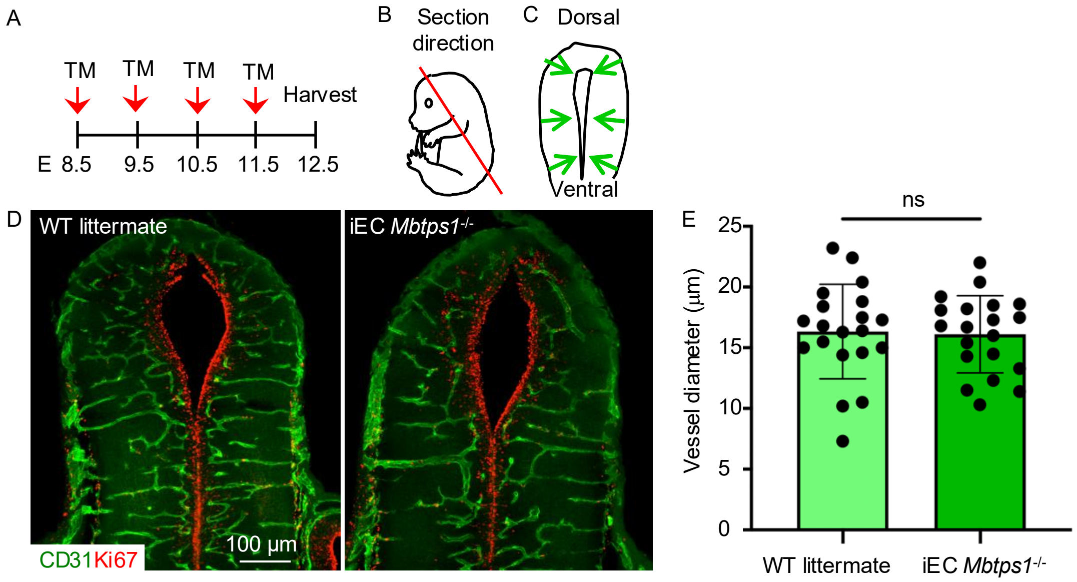

Supplemental Figure 2. Relatively normal blood angiogenesis in iEC *Mbtps1*$^{-/-}$ mice. A. Tamoxifen-induced S1P deletion strategy in iEC *Mbtps1*$^{-/-}$ mice. **B**. A diagram depicting the sagittal section of an embryo (E12.5). **C**. A diagram illustrating the directional growth of blood vessels toward the subventricular zone at E12.5 on the sagittal section. **D**. Confocal images of blood vasculature in the developing neural tissues at E12.5. CD31: blood vessels, Ki67: proliferating radial glial cells. **E.** Quantification of vessel diameters in the E12.5 brain by ImageJ. n=15 images/genotype. Data represent at least three experiments. The graphs were plotted as Mean ± SD. An unpaired t- test was performed for the statistical analysis. ns: not significant.

Supplemental Figure 3

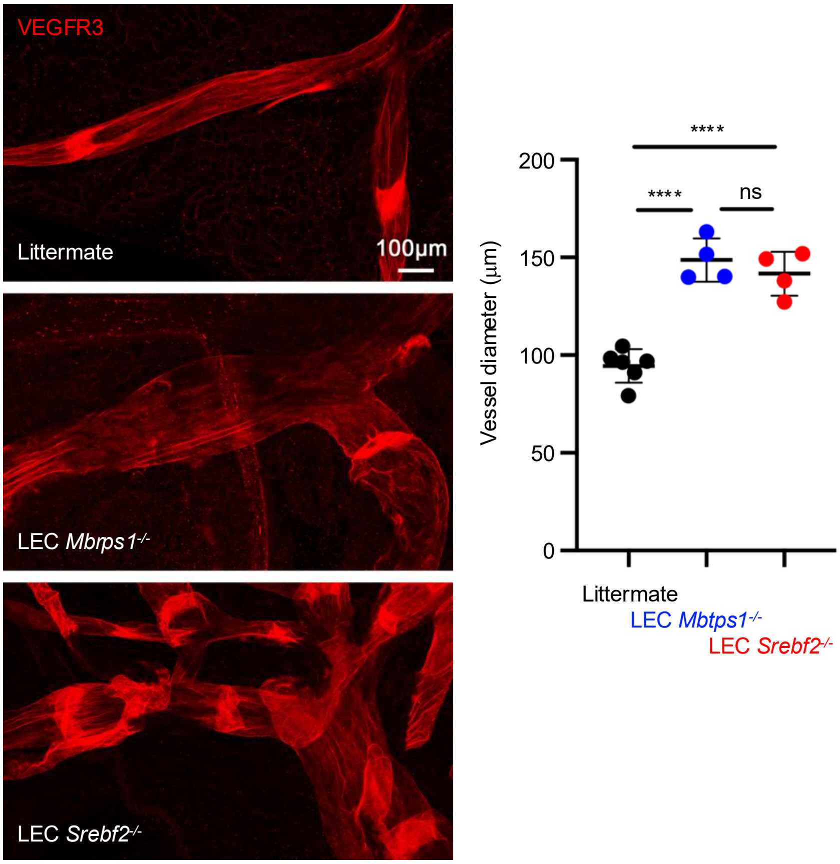

Supplemental Figure 3. Mice with lymphatic endothelial cell-specific MBTPS1 or SREBP2 deletion show enlarged mesenteric collecting lymphatic vessels. Representative whole-mount IF images of mesenteric lymphatic vessels (red) of littermate controls, LEC *Mbtps1*$^{-/-}$ and LEC *Srebf2*$^{-/-}$ mice (20 days of age). The diameter of the vessels are significantly increased in the mutant mice compared with that in the controls. n=6 for littermate controls; n=4 for each mutant genotype, respectively. 4 mesenteric collecting lymphatic vessels from the jejunum section of each animal were analyzed. Each dot represents one mouse on the graph. The graphs were plotted as Mean ± SD. One way ANOVA was performed for the statistical analysis. ns: not significant. ****p<0.0001.

Supplemental Figure 4

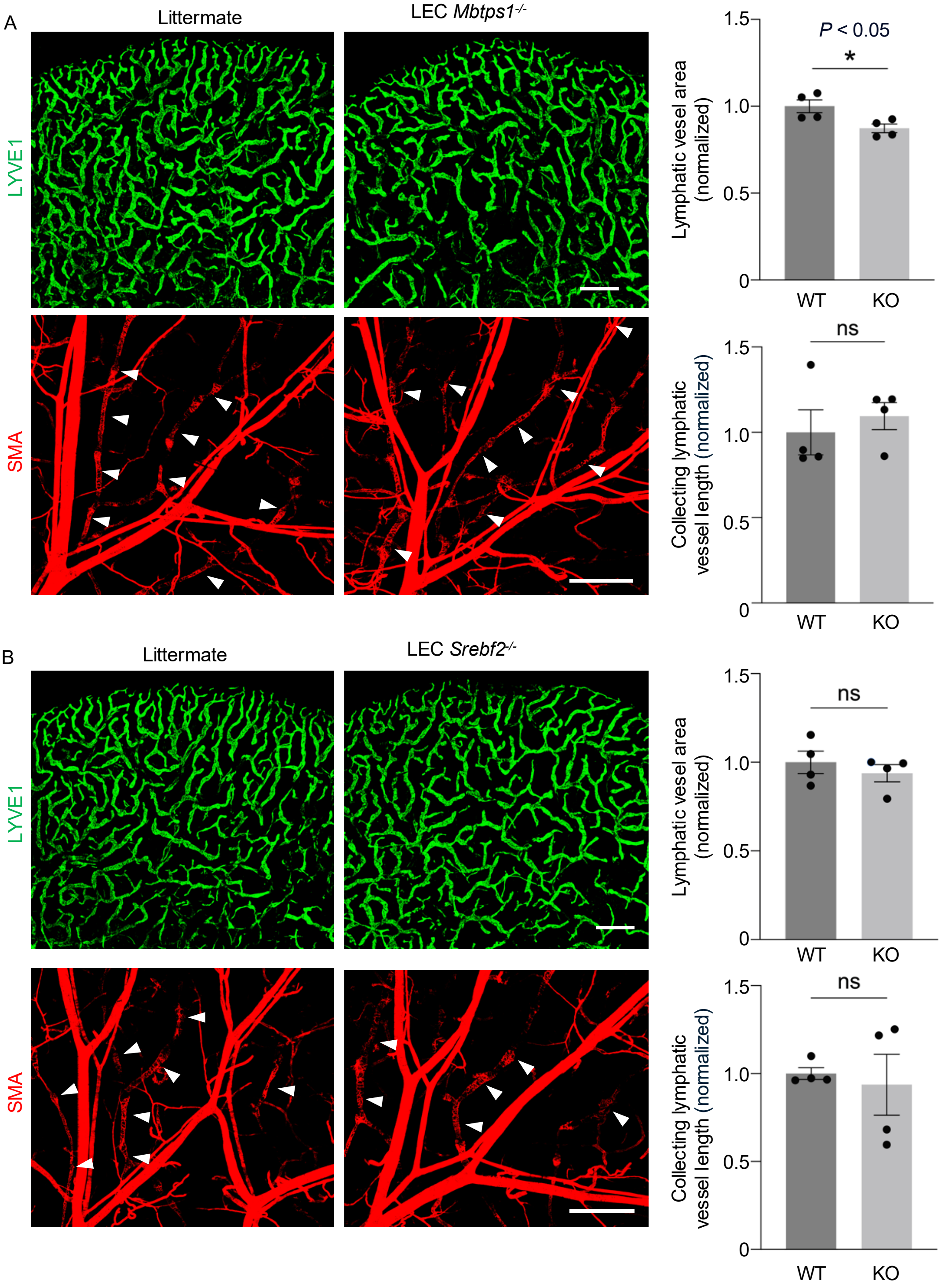

Supplemental Figure 4: Effect of loss of LEC *Mbtps1* or *Srebf2* on lymphatic vessel development in the postnatal ear skin. A. Up left, representative stitched images of the dorsal ear skin of wildtype littermate (WT) and *Mbtps1* knockout (KO) mice stained for LYVE1; Up right, quantification of the lymphatic microvessel area of the dorsal ear skin standardized to wildtype mice. Lower left, representative stitched images of the dorsal ear skin of wildtype and *Mbtps1* knockout mice stained for α-smooth muscle actin (SMA). White arrows denote SMA-positive collecting lymphatic vessels; Lower right, quantification of the collecting lymphatic vessel length of the dorsal ear skin standardized to wildtype mice. **B.** Up left, representative stitched images of the dorsal ear skin of wildtype (WT) and *Srebf2* knockout (KO) mice stained for LYVE1; Up right, quantification of the lymphatic microvessel area of the dorsal ear skin standardized to wildtype mice. Lower Left, representative stitched images of the dorsal ear skin of wildtype and *Srebf2* knockout mice stained for SMA. White arrows denote SMA-positive collecting lymphatic vessels; Lower right, quantification of the collecting lymphatic vessel length of the dorsal ear skin standardized to wildtype mice. Scale bar, 100 µm. n = 4 ears per group. Data represent mean ± SEM, Unpaired t-test with Welch's correction. ns: not significant.

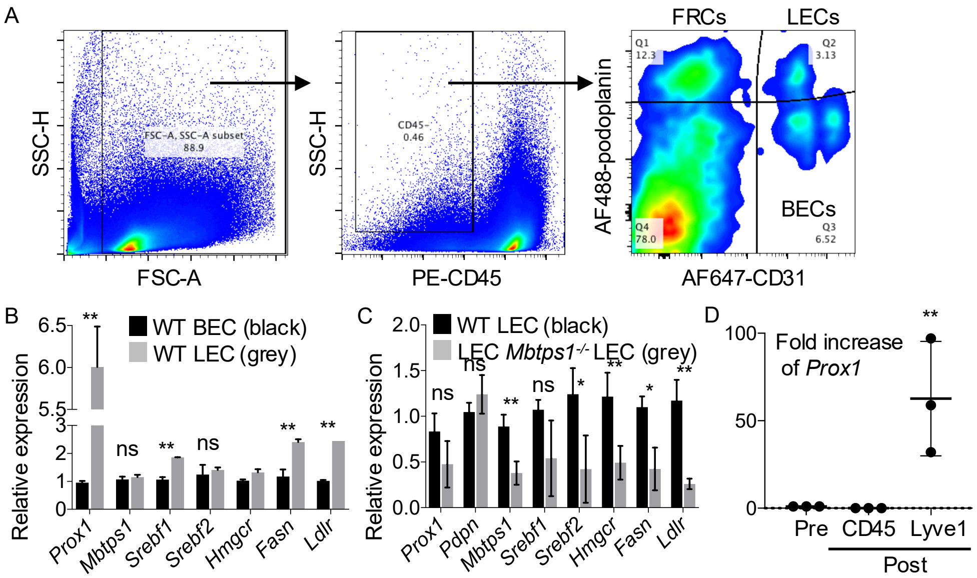

Supplemental Figure 5. Reduced lipogenic gene expression in the lymphatic endothelial cells of LEC *Mbtps1*$^{-/-}$ mice. A. Cell sorting strategy of BECs and LECs from the lymph nodes of adult mice (6 weeks) LECs; lymphatic endothelial cells, BECs; blood endothelial cells. **B**. Differential expression of lipogenic genes between WT BECs (black) and WT LECs (grey) (N = 3 in each genotype). **C**. Reduced lipogenic gene expression in WT LECs (black) and LEC *Mbtps1*$^{-/-}$ LECs (grey). Data represent mean ± SEM, Unpaired t- test was performed for the statistical analysis. ns: not significant (N = 3 in each genotype). **D**. Magnetic cell separation efficiency of Prox1^{+} LECs from embryonic skin using anti-Lyve1-beads for qRT-PCR (N = 3). Data represent at least three experiments. The graphs were plotted as Mean ± SD. One way ANOVA was performed for the statistical analysis. *P<0.05, **P<0.01.

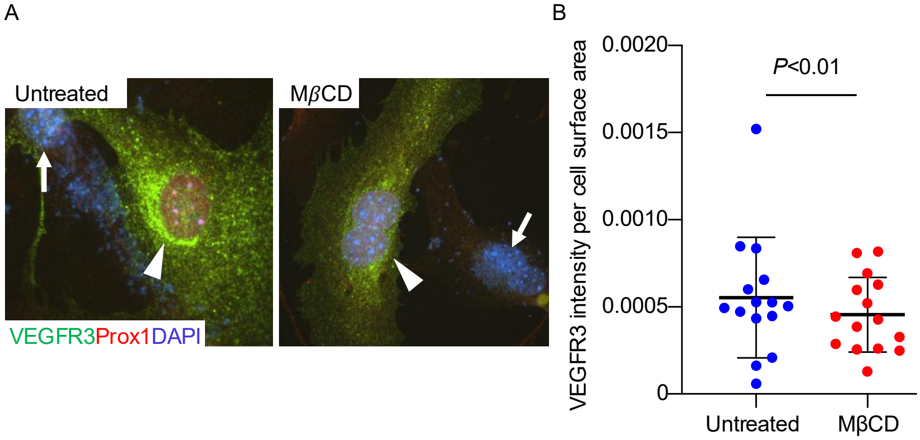

Supplemental Figure 6. Methyl-*β*-cyclodextrin treatment reduced VEGFR3 in cultured hLECs. **A.** Immunofluorescence of VEGFR3 and Prox1 in cultured human LECs. LECs were treated with methyl-*β*-cyclodextrin (30 min) before staining. Arrowhead indicates LECs, Arrow indicates non-LECs. **B.** Quantification of VEGFR3 signal intensity per cell surface area in **A** was performed using ImageJ software (n = 15). Data represent mean ± SD, unpaired t-test with Welch's correction was performed for the statistical analysis.

Supplementary Movie 1: 3D view of small intestinal lacteal and submucosal lymphatics of the littermate control.

Supplementary Movie 2: 3D view of small intestinal lacteal and submucosal lymphatics of LEC *Srebf2*$^{-/-}$ mouse.
