# Supplementary material for: Site-1 protease–mediated cholesterol metabolism is essential for lymphatic development in mice
Source: JCI Insight. 2025 Oct 22;10(20):e188637. doi: 10.1172/jci.insight.188637 (PMC12581667; doi:10.1172/jci.insight.188637)
Supplement: Unedited blot and gel images [file jciinsight-10-188637-s126.pdf]

Full unedited gel for Fig. 6A

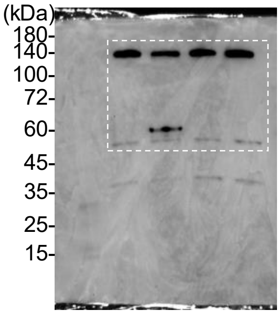

IB: anti-SREBP2

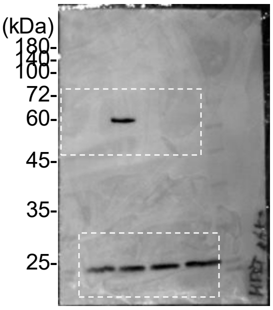

IB: anti-phosphor Akt and HPRT

Full unedited gel for Fig. 6C

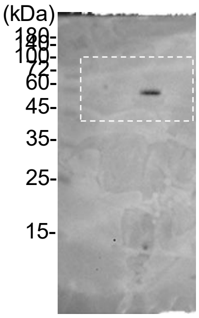

IB: anti-phospho-Akt

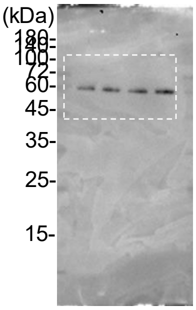

IB: anti-Akt

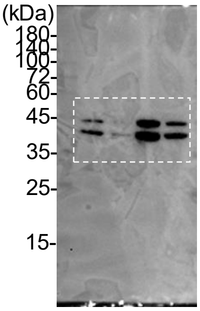

IB: anti-phospho ERK1/2

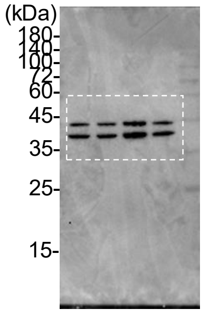

IB: anti-ERK1/2

Full unedited gel for Fig. 6D

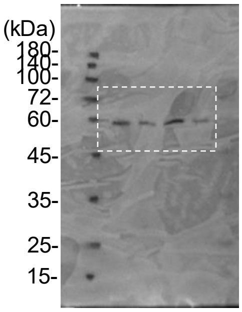

IB: anti-phospho-Akt

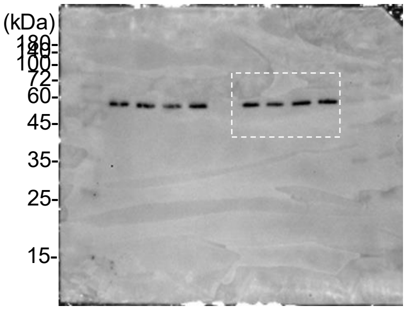

IB: anti-Akt

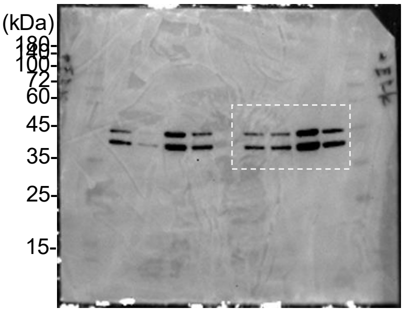

IB: anti-phospho-ERK1/2

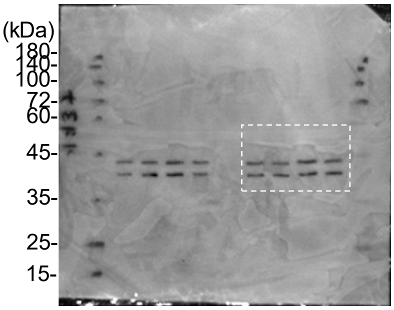

IB: anti-ERK1/2

Full unedited gel for Fig. 6E

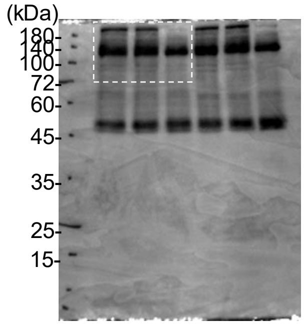

IB: anti-VEGFR3

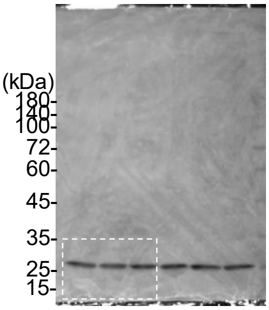

IB: anti-HPRT

Full unedited gel for Fig. 6G

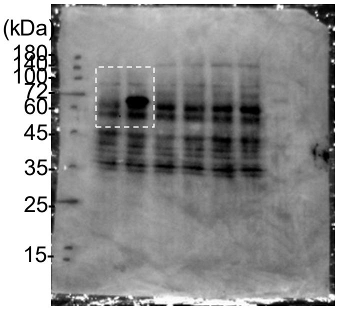

IB: anti-Myc

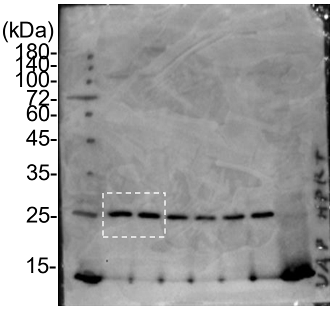

IB: anti-HPRT

Full unedited gel for Fig. 6I

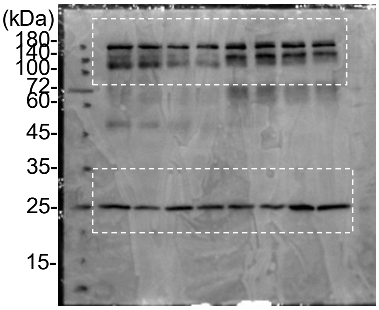

IB: anti-VEGFR3 and HPRT
